# A review of interventions addressing structural drivers of adolescents’ sexual and reproductive health vulnerability in sub-Saharan Africa: implications for sexual health programming

**DOI:** 10.1186/1742-4755-11-88

**Published:** 2014-12-13

**Authors:** Joyce Wamoyi, Gerry Mshana, Aika Mongi, Nyasule Neke, Saidi Kapiga, John Changalucha

**Affiliations:** National Institute for Medical Research, P.O Box 1462, Mwanza, Tanzania; Mwanza Intervention Trials Unit, P.O Box 11936, Mwanza, Tanzania; London School of Hygiene and Tropical Medicine, Keppel Street, London, WC1E 7HT UK

**Keywords:** Structural drivers, Structural interventions, Young people, Sexual and reproductive health, Adolescent SRH programming

## Abstract

**Background:**

Young people particularly women are at increased risk of undesirable sexual and reproductive health (SRH) outcomes. Structural factors have been reported as driving some of these risks. Although several interventions have targeted some of the structural drivers for adolescent’s SRH risk, little has been done to consolidate such work. This would provide a platform for coordinated efforts towards adolescent’s SRH. We provide a narrative summary of interventions in sub-Saharan Africa (sSA) addressing the structural drivers of adolescents’ SRH risk, explore pathways of influence, and highlight areas for further work.

**Methods:**

33 abstracts and summary reports were retrieved and perused for suitability. Fifteen documents met the inclusion criteria and were read in full. Papers and reports were manually reviewed and 15 interventions that met the criteria for inclusion were summarised in a table format.

**Results:**

Most of the interventions addressed multiple structural factors, such as social norms, gender inequality, and poverty. Some interventions focused on reducing economic drivers that increased sexual risk behaviours. Others focused on changing social norms and thus sexual risk behaviours through communication. Social norms addressed included gender inequality, gender violence, and child socialisation. The interventions included components on comprehensive sexuality and behaviour change and communication and parenting, using different designs and evaluation methods. Important lessons from the narrative summary included the need for a flexible intervention design when addressing adolescents, the need for coordinated effort among different stakeholders.

**Conclusion:**

There are encouraging efforts towards addressing structural drivers among adolescents in (sSA). There is, however, a need for interventions to have a clear focus, indicate the pathways of influence, and have a rigorous evaluation strategy assessing how they work to reduce vulnerability to HIV. There is also a need for coordinated effort among stakeholders working on adolescent vulnerability in sSA.

**Electronic supplementary material:**

The online version of this article (doi:10.1186/1742-4755-11-88) contains supplementary material, which is available to authorized users.

## Background

Sub-Saharan Africa (sSA) continues to bear the brunt of the Human Immuno-deficiency Virus (HIV) epidemic, with two thirds of those infected residing in the region[1]. Women and girls are the most affected accounting for 60% of the people living with HIV from the region. Young people aged 15-24 years account for an estimated 45% of new infections globally. In this age group, women aged 15-24 years are eight times more likely to be infected than young men (ibid), pointing to the urgent need for prevention efforts to focus on girls to abate the epidemic.

Young people’s sexual behaviour is influenced by their social and economic context. Aspects of this context that increase or decrease susceptibility of young people to these outcomes include: gender issues in relationships and families, social norms and poverty[2, 3]. Most families in rural sSA are poor[4]. Studies in Tanzania[5, 6] have noted that in order to satisfy their material needs, young women from poor families may engage in transactional sexual activity with multiple partners or casual partners or agree to have sex without a condom. Desmond *et al.,*[7] found that in an endeavour to maximise financial gains from sex, women engaged in high risk sexual practices such as anal sex.

Several approaches have been employed to address the situation of HIV in young people. These comprise individual (biomedical and behavioural) approaches as well as those that go beyond the individual. Published literature on sexual health interventions have emphasised the importance of changing young people’s risk profiles (especially their knowledge level and attitudes), but have consistently failed to produce long-term behaviour change or improved sexual health outcomes at the population level[8–11]. For example, their emphasis has been on assessing individual level risk factors such as contraception and condom use knowledge. As much as unwanted pregnancy and STIs pose serious problems to young people, an exclusive reliance on “risk factor” explanation enhances the likelihood that our understanding of these problems is denuded of social meaning[12] and that interventions to address such issues remain focused exclusively on reducing adolescent risk behaviour rather than understanding the social environment facilitating risk.

There have been several suggestions concerning HIV prevention efforts moving beyond individualistic approaches towards new approaches that engage with underlying socio-structural drivers of patterns of practices that influence vulnerability to HIV infection[13–16]. Structural drivers of HIV/AIDS have been conceptualised as ‘physical, social, cultural organizational, community, economic, legal or policy aspects of the environment that influence the risk and vulnerability environment and thus acting as barriers to, or facilitators of, HIV prevention and treatment behaviour’[14, 17]. Causal pathways link structural factors (social, economic, political, and environmental factors) and risk of HIV. Efforts to address these underlying factors are commonly referred to as structural approaches and seek to change the root causes or structures that affect individual risk and vulnerability to HIV[16]. Hence, structural approaches to HIV prevention recognise the limitation of the biomedical/behavioural paradigm, and take a broader approach to the nature of transmission, arguing that “HIV prevention behaviour is affected by the environment as well as by the characteristics of individuals at risk”[18].

There is limited published work summarising interventions addressing structural drivers for young people’s SRH risk from sSA. We aimed to fill this gap by conducting a review and summary of such interventions among young people aged 14-24 from sSA. We further explore pathways of influence, reflecting on intervention strengths and weaknesses and lessons learnt highlighting areas for further work. This review provides information that may be helpful in the identification of gaps for future young people’s SRH programming.

## Methods

Published studies were found by searching the following electronic databases: web of knowledge, PubMed, International Bibliography of Social Sciences, and Google scholar. A combination of search terms were used, including: sSA; adolescent and/or young people interventions, adolescent and/or structural interventions, transactional sex; sexual behaviour; HIV risk, HIV/AIDS, HIV prevention; adolescents; young women; adolescent health; adolescent and/or reproductive health; cash transfers; adolescent and young people livelihoods; adolescent and/or young people poverty; norms; and parental socialisation.

Unpublished studies and non-indexed reports were sought through searches using Google Scholar, targeted searches of websites of relevant organisations. Key scholars and interventionists at LSHTM, UNDP, UNICEF, and several non-governmental organisations (NGOs) were also approached and asked to recommend interventions. Examples of the NGOs contacted were: populations council, International Centre for Research on Women, Global giving and TIOS. We only searched for English language papers.

All searches were for articles dating from 2000 to 2013. The scope of the review was limited to interventions conducted in sSA that attempted to tackle gender norms or inequities and livelihoods or poverty and were aimed at vulnerable young people (aged 14-24 years). The interventions included had either HIV or HIV risk outcomes (such as condom use, multiple partners, intimate partner sexual violence, intergenerational sex and STIs) and broader behaviour change and interaction skills. Interventions were only included if they reported atleast one outcome. Due to the small number of interventions identified, interventions were included whether or not they reported outcomes in peer reviewed journals and some that were not exclusively focused at young people but also age groups beyond 24 years.

Titles and abstracts of studies identified through electronic searches were reviewed to determine whether they met the inclusion criteria. Most of the articles were excluded based on the title alone. Thirty three abstracts and summary reports were retrieved and Thirty three abstracts and summary reports that met the inclusion criteria were retrieved and screened by two researchers in order to select potentially relevant articles and reports. When summaries were judged to have met inclusion criteria, full length texts were then reviewed. Where the two researchers disagreed on relevance of the retrieved article a consensus was reached by consulting other co-authors who are very experienced in the subject area. Fifteen publications were retained after meeting the inclusion criteria, five of which were derived from our search of grey literature.

For all the 15 studies, data were manually compiled describing the following features: aim of the intervention, intervention design, target group and sample size, duration of the intervention, outcomes including those related to HIV. Owing to significant differences in the populations studied, settings, outcomes, data analyses and reporting of included interventions, no attempts were made to combine the data in a meta- analysis.

Due to a limited number of interventions addressing our objective, studies were selected to reflect different designs and hence the nature of studies reviewed were not reviewed to address general guidelines of research quality and risk of bias as described according to Jaeshcke[19] criteria guidelines. Publication bias was therefore not assessed in the current review.

The interventions were reviewed and grouped according to the following themes: economic empowerment of women, economic empowerment plus school attendance, gender empowerment and safe spaces, comprehensive sexuality and behaviour change communication, and parental socialisation. These categories were subsequently used to structure the presentation of the results. It is worth highlighting that several interventions presented findings that fitted into more than one category.

## Results

A total of 33 interventions were reviewed. Fifteen interventions met the selection criteria and are included in the summary here. Eight of the interventions were evaluated quantitatively while five used a combination of qualitative and quantitative approaches. Two interventions were evaluated mainly by qualitative approaches. The interventions were conducted in both rural and urban settings and participants were recruited both from schools and within communities. The age range of the intervention participants was 10-44 years, with two interventions having participants who were older than 24 years. Over half of the interventions (8/15) have an explicit focus on girls. Nine of the interventions had their results published in peer review journals. The rest were published in reports.

An overview of the interventions included in this review describing their primary and secondary aims is presented in Table 1.

**Table 1:** **Descriptions for iterventions for young people**

Intervention/country	Aim	Intervention method/design & duration	Target group &Sample size	Outcomes/results
**Economic empowerment of women**				
1) Shaping the Health of Adolescents in Zimbabwe (SHAZ!) Program[20]	Increased knowledge, Increased economic empowerment, Reduced inter-generational TS	**Pilot study**	- 50 poor orphaned, out-of-school, girls aged 16-19 years	- Increase in HIV-related knowledge and relationship
		- Uncontrolled study for 6 months		- Power, no significant change in current sexual activity or condom use at last sex
		- Microcredit loans	- Living on the	- Increased relationship power[21]
		- Business skills training	outskirts of Harare, Zimbabwe	- Increased HIV risk through new mobility and economic strategies
		- Mentorship	- 315 aadolescent girls, orphans, average age 18	- Increase in HIV-related knowledge and relationship power, no significant change in current sexual activity or condom use at last sex
		**Phase II study:**		
		Randomized clinical trial (RCT)		
		Study		- Decrease in food insecurity
		Duration 24 months, Adaptation of Stepping Stones, including expanded training including negotiation skills, Integrated social support		- Increase in equitable gender norms
				- Physical and sexual violence reduce by 58% over a 2-year period
		Access to HIV and reproductive health services		
2) Intervention with Microfinance for AIDS & Gender Equity (IMAGE)[22, 23], South Africa	Reduced HIV risk behaviour	Cluster randomised trial, duration of 3 year	- A sample of 430 poor women aged 14-35 years identified through participatory wealth ranking	- 55% increase in experience of IPV after 1 year
				- Increase in HIV knowledge, communication, testing & risk reduction
				- 32% reduction in communication with household members to young people in households
				- Greater involvement in collective action and social groups
				- No impact on HIV incidence in wider community
				- No difference in unprotected sex at last occurrence with non-spousal partner in past 12 months
				- 11% increase in condom use
				At last sex
		- Microfinance (individual borrowing and repayment of loans over 10 or 20 week cycles)		
		- Participatory learning and action curriculum integrated into loan meetings (10 training sessions done within centre meetings every 2 weeks (approx. 6 months)) Community mobilization for 6 to 9 months following initial training		
		- HIV prevention education		
3) The Tap & Reposition Youth (TRY)[24, 25], Kenya	Increased reproductive health & HIV knowledge Increased sexual negotiation skills Increased Income & savings	Pre-test, post-test design, with matched comparison (222 pairs), length of participation ranged from <1 year (n = 71), 1 to 2 years (n = 81) and 2 to 3 years (n = 70)	- A total of Out-of-school females aged 16-22 years	- Increase Savings
		- Group-based microfinance loans, Livelihoods skills training		
		- RH & HIV prevention training		
				- Increase in liberal attitudes towards gender roles
			- Living in low income & slum areas of Nairobi	- 1.7 times more likely to refuse sex than girls in control group
				- 3 times more likely to insist on condom use than girls in control group
4) Incentivising safe sex: a randomised trial of conditional cash transfers for HIV and sexually transmitted infection prevention in rural Tanzania[26]	To evaluate the use of conditional cash transfers as a HIV and sexually transmitted infection prevention strategy to incentivise safe sex	An unblended, individually randomised controlled trial	- A sample of 2399 persons aged 18-30 years	- High value CCT arm v.s. controls: adjusted RR = 0.073 (95% CI 0.47-0.99)
		- Intervention arms: low value conditional cash transfer v.s., high value conditional cash transfer		- High value CCT arm v.s., low value CCT arm: RR = 0.76 (95% CI0.49 -0.92)
				- Significant reduction in the combined point prevalence of four curable STIs among high value CCT arm
		- Tested participants every 4 months over a 12 months period for the presence of common STIs		
5) Survival skills training for orphans (SSTOP)[27], Mozambique	To reduce transactional sex	Intervention:	- Females aged 14-19 years	Qualitative & anecdotal evidence found:
			- Responsible for	- Increased financial organization
		Income generating skills	caring for younger siblings, & other disadvantaged girls	- Increase vocational skills - Reduction in early marriage
				- Increased economic empowerment
		- Girls aged 9-13 learned to make soap, candles, sewing, or knitting		- Reduction in early sexual activity without protection
		- Girls aged 14-19 attended sewing classes; HIV prevention education; & gender training including legal protection for women		
6) Creating futures[28] (Durban, South Africa)	Objective 1:	Pilot intervention combining Stepping Stones and Creating Futures	- Piloted in urban informal settlements in with 232 young people (110 men, 122 women)	**Objective 1:**
	To strengthen young people’s livelihoods and economic power through reflection and action			
				- Livelihoods improved for women and men after the intervention
		- The study design was an interrupted time-series design, with baseline measures at zero and two weeks and follow-ups at six and 12 months post-baseline.		
			- Average age of 21.7 years	
				- Mean earnings in the past month increased over the 12 months. For women this increased from US$14 at baseline to US$49 (a 345% increase (p < 0.0001)) at 12 months and for men from US$36 at baseline to US$104 (a 283% increase (p < 0.0001)) at 12 months
	Objective 2: Aimed to reduce women’s experience and men’s perpetration of physical or sexual IPV			
		- Consisted of livelihoods and economic power intervention involving 21 sessions of three hours, delivered by trained peer facilitators		
				**For objective 2:**
				Women reported a statistically significant reduction in their experience of sexual or physical IPV in the past three months from 29.9% at baseline to 18.9% at 12 months (a 37% reduction (p < 0.046)
				- Women’s experience of sexual IPV also declined significantly from 11.1% at baseline to 3.6% at 12 months (p < 0.018)
				- Men’s perpetration of physical or sexual IPV in the past 3 months, while declining from 25% to 21.9% (a 23% reduction) was not statistically
				Significant
**Economic empowerment plus school attendance**				
7) Zomba cash transfer[29], Malawi	Increased income Increased EducationReduce HIV risk	Randomised control trial, 2 years Cash transfers (CTs) conditional and on regular school attendance v.s. unconditional CTs (average amount US $10)	A sample of 1289 never married girls aged 13-22 years in 176 enumeration areas in Zomba	**One-year follow-up:**
				- Reduced onset of sexual activity by 31.1%
				**At 18 months follow-up:**
				- Intervention group had 64% reduction in HIV prevalence and 76% reduction in HSV-2 prevalence
				- Reduced age of partners in those in intervention
				- No significant differences between conditional and unconditional intervention group, although the study was not powered to show this
8) Western Kenya schooling intervention[30]	To reduce HIV incidence in schools	Randomised control trial, 4 years, Comparing 4 school-based HIV/AIDS interventions:	A sample of 70,000 school boys and girls in school	Teacher training:
				- No impact childbearing
				- Increase in HIV knowledge If pregnant, more likely to be married
		- Training teachers in HIV/AIDS curriculum		Critical thinking:
				- Increase knowledge & condom use
		- Critical thinking on role of condoms		- No impact on sexual activitySchool uniforms:
				- Reduction in dropout rates 17% in boys, 14% in girls
		- Reducing the cost of education by providing school uniforms		
				- Reduction in teen marriage 9% in girls
				- Reduced childbearing 12%Relative risks:
		- Relative risk campaign		
				- Reduction in childbearing 28%
				- Increased sexual activity in boys
				- No impact on pregnant teen couples
				- Reduction in cross-generational pregnancies 61%
**Gender empowerment and safe spaces for young people**				
9) Binti Pamoja Centre (Daughters United centre)[31, 32], Kenya	Create safe spaces for girls to reduce: violence, Female genital mutilation, Sexual abuse, Rape, Prostitution Poverty and Increase: Reproductive health knowledge, Financial education, Leadership & personal skills	Community intervention:	Girls aged 11-18 living in the Kibera slum	**2002 to present**
		- Sampled adolescents from 4 ethically distinct villages in Kibera		- Baseline data highlights social isolation for many girls & 55% of girls live with neither or only one parent
		- Mapped all safe spaces in the community		
				- >30 safe spaces established reaching >1000 girls
		- Used photography, drama, writing & group discussion		
				- Positive changes in social networks, mobility & gender norms
				- Increased financial literacy, banking services usage, savings, & communication with parents/guardian on financial issues
		- Peer education & empowerment workshops		
		- Developing skills in budgeting, savings, setting financial goals		
				- Increased confidence & positive self-esteem
		- Provided educational scholarships		
10) Siyakha Nentsha[33], South Africa	A life-orientation program to improve lifelong skills & well being of young people	Quasi-experimental, control arm, 18 month follow-up, 4 years Three study arms:	Boys & girls aged 14-16 in schools	- Increased autonomy for girls in how they spend their money & control their lives
				- Increased HIV related knowledge
				- Young men had reduced onset of sexual activity and fewer partners
		- SRH/HIV, social support, financial education		
		- SRH/HIV & social support		
		- Delayed Intervention (i.e. control group)		
11) ICRW Vitu Newala[34], Tanzania	Understand specific vulnerabilities of adolescent girls and empower them, increase girls positive attitudes and beliefs on girls’ social protection	Pilot project Qualitative assessments throughout:	Adolescent girls	- Video parlours, discos & traditional initiation ceremonies identified as places where girls felt unsafe
		- Repeating the same participatory learning activities,		- Community put in place laws & changed practices to provide social protection
		- Series of IDIs with young people,		
		- An evaluation workshop		
**Comprehensive sexuality and behaviour change communication**				
12) Soul City Institute for health & development[35], South Africa	Increase: social change, Social mobilization, Advocacy and reduce HIV incidence	Promoting health & social change via TV, radio, & print Soul Buddyz:	Soul buddyz:	Soul City & Soul Buddys exposure
		- Spin off of Soul City TV series using edutainment	- Children aged 8-14 years, their teachers & their caregivers	- Increased: Self-perceptions on risk, Resistance to peer pressure
		One love:		
			- One love: Adults	
				- Statically significant shifts in social norms, especially sexual norms
				- Reduced Perception of
		- Challenged social norms on multiple & concurrent partnership		women’s dependence on men (68% vs. 61%, p < 0.05)
13) Stepping Stones[36, 37], South Africa	Increase: Sexual health knowledge, Communication skills, Ccritical reflection and reduce Sexual health risk	Cluster randomised controlled trial, 2 years	A sample of 1077 HIV negative Persons aged 15-26 years, mostly attending school	- HIV IRR = 0.85 (95%CI: 0.60, 1.20; p = 0.35)
				- HSV2 IRR = 0.69 (95%CI: 0.47, 1.03; p = 0.07)
		- 70 villages randomized to either 13 3-hour sessions and 3 peer group meetings, or a 3-hour session on safer sex and HIV.		
				- Men’s disclosure of perpetrating severe Intimate partner violence reduced at 12 & 24 months (p = 0.11 & p = 0.05)
				- Reduced Problem drinking among men
**Parenting and socialisation**				
14) Families Matter! (FMP),[38, 39], Kenya	Reduce age at first sex and increase ppositive parenting practices	Community-based intervention using parent-child dyads, 2 years (2004-2006)	375 Parents/carers of 10-12 year-olds	- Increased Parenting skills & communication about sexuality & risk reduction
		Five consecutive 3-hour sessions on sexual risks and effective parent-child communication		- Parents’ attitudes regarding sexuality education changed positively.
				Five of the six composite parenting scores reported by parents, and six of six reported by children, increased significantly at 1 year post-intervention.
15) Mema kwa Jamii (Good Things for Communities, *MkJ)*,[40, 41], Tanzania	Reduce SRH risks in youth through improved parenting	Community-based pilot parenting intervention, 2007-2010	Approximately 1355 parents of young people aged 10-18 years	Qualitative indications of impact on:
				- Parents socialised their male children differently from female
		Opinion leaders in four communities trained to training peer parents on parenting following diffusion of innovation theory over a period of 1 year		
				- Improved parent-child relationships and collective efficacy

With regards to measuring effects of the intervention, different statistical tests were used for various studies. Some authors provide a computation of the effect size for the differences between groups and some provide Odds Ratios, confidence intervals and p-values. On the other hand, some studies provided descriptive statistics which offered a perspective on the type and size of difference[29, 30], while others reported positive improvements in their outcomes of interest e.g. increased autonomy, increased parent-child communication but no statistics were provided.

Most of the interventions seemed to have more than one focus. Seven of the interventions had some focus on HIV prevention through changing gender norms, improving school attendance, improving economic situation of participants’ and creating safe spaces. Eight of the interventions focused on either economic empowerment alone or economic empowerment and school attendance. Three focused on livelihoods and safe spaces, three on comprehensive sexuality and behaviour change and communication, while two took a parenting perspective.

### Interventions with a focus on economic drivers of SRH risks

#### Economic empowerment of women

Six of the interventions addressed economic empowerment of women. These were: Shaping the Health of Adolescents in Zimbabwe (SHAZ!)[20], Intervention with Microfinance for AIDS and Gender Equity (IMAGE)[42], The Tap and Reposition Youth (TRY) (Kenya)[24, 25], incentivising safer sex[26], and the combined stepping stones and creating futures intervention[28] and Survival skills training for orphans (SSTOP)[27].

SHAZ! and TRY targeted women aged 16-24 years while IMAGE looked at the age group 14-44. The most common characteristic for the girls in the interventions was that they were economically inactive due to orphanhood, living in low income environments.

SHAZ!, IMAGE, and the incentivising safer sex interventions were evaluated using a randomised controlled trial design. TRY used evaluations that were purely qualitative.

The intervention on incentivising safer sex used conditional cash transfers as a HIV/STI prevention strategy among both men and women aged 18-30 years, while SSTOP focused at improving income generation skills among 14-19 year old young women.

Overall, the interventions report positive impact. A notable positive result across the interventions was the increased knowledge in reproductive health and improved financial management skills. The combined creating futures and stepping stones intervention targeted young women and men of average age 21 years[28]. The intervention aimed to strengthen young people’s livelihoods and economic power through reflection and action and to reduce women’s experience and men’s perpetration of physical or sexual IPV. The programme was evaluated using quantitative and qualitative methods.

For the two (e.g. IMAGE and SHAZ!) interventions that focused on HIV as one of the outcomes, both point to little impact on HIV incidence. Interestingly, one of the intervention’s activities (SHAZ!) had unforeseen consequences in that it increased young women’s risk to HIV. It was noted that as young people struggled to repay the loan, they adopted risky strategies.

IMAGE, incentivising safer sex intervention, combined stepping stones and creating futures projects presented statistical results at the time of this review. At 1-year follow-up, the communities receiving the IMAGE intervention saw a 55% reduction in IPV as compared to the control communities. The IMAGE intervention also increased HIV knowledge, communication, testing, and risk reduction among young women who participated in the intervention as compared to those in the control communities[22]. Although IMAGE also sought to reduce HIV incidence in the wider community where the interventions occurred, there was no difference in HIV incidence between the observed communities at the time of analysis.

At the end of 12 months, the results from incentivising safer sex intervention, showed significant reduction in the combined point prevalence of the four curable STIs tested every 4 months in the incentivized group that was eligible for the $20 payments and no such reduction was found for the group receiving the $10 payments[26]. De Walque et al.,[26] conclude that their results suggest that conditional cash transfers used to incentivise safer sexual practices are a potentially promising new tool in HIV and STIs prevention.

The stepping stones and creating futures intervention improved livelihoods for both men and women[28]. Women reported a statistically significant reduction in their experience of sexual or physical IPV in the past three months from 29.9% at baseline to 18.9% at 12 months (a 37% reduction (p < 0.046)). Although men’s perpetration of physical or IPV in the past 3 months declined from 25% to 21.9% (a 23% reduction) that was not statistically significant[28].

#### Economic empowerment plus school attendance

Interventions on economic empowerment of women also aimed at reducing structural barriers to education by increasing school attendance of young women and thereby decreasing their HIV risk. Both interventions in this category utilised a randomised control trial design. These interventions include: the Zomba cash transfer[29]; and the Western Kenya School Uniform project[30].

Both trials increased school attendance. The Zomba cash transfer intervention found a 60% reduction in HIV incidence in 18 months and 75% lower prevalence of HSV-2 in the treatment group[29]. The Western Kenya schooling intervention reported overall positive results on variables such as: reduced dropout rates (17% for boys and 14% for girls); reduced teen marriages among girls (9%); improved HIV knowledge; and 61% fewer intergenerational sexual relationships[30].

### Interventions with a focus on the social norms

The social norms that the interventions focused on were those in relation to gender inequality, gender violence and norms on child socialisation.

#### Gender empowerment and safe spaces for young people

Binti Pamoja Centre[31, 32], Siyekha nentsha[33], and Vitu NEWALA project[34] focused on gender empowerment and safe spaces. At the time of the review, the results of all the three interventions were not presented in peer reviewed journals but in reports.

Binti Pamoja baseline results highlighted social isolation for many girls with 55% living with neither or only one parent[31, 32]. As such, over 30 safe spaces were created by trained community mentors and reached over 1000 girls in the last 3 years. Process monitoring results showed that intervention participants had increased confidence and positive self-esteem, and that the community perceived the intervention positively. There were also positive changes in young people’s social networks and mobility, gender norms, financial literacy, use of bank services, saving behaviour, and communication with parents/guardian on financial issues.

Siyekha Nentsha’s final results are not yet available, but preliminary results suggest a number of positive changes such as young women reporting increased autonomy in how they spend their money and a wider sense of ability to take control of their own lives[33].

Vitu Newala’s pilot intervention results showed that it empowered young women who participated and the attitudes and beliefs of individual boys and adults in the community who took part have begun to change. In particular, girls identified video parlours, discos, and traditional initiation ceremonies as places where they felt more at risk. While the project did not set out to intentionally change rules governing video parlours and discos, or to change practices around the traditional initiation ceremonies, the conversation that ensued from Vitu Newala led to decisions within communities to put in place by laws and change practices in order to protect young women.

#### Comprehensive sexuality and behaviour change communication (BCC)

Girls Power Initiative[43], Soul Buddyz and One Love campaign by Soul City Institute[35] and Stepping Stones[44] focused on comprehensive sexuality and behaviour change communication strategies. Although all the three interventions focused on information provision for social change and activities included information provision through mass media, social mobilization, advocacy, and through participatory activities, only Stepping Stones evaluated and presented results in a peer reviewed publication.

Stepping Stones indicated a 34.9% (incidence rate ratio 0.67,95% CI 0.46-0.97) reduction in the incidence of HSV-2 among participants, a reduction in the proportion of men reporting they committed intimate partner violence, and the proportion of men reporting transactional sex[44]. Other positive results include reduction in problem drinking among men, hence, changes in drinking norms.

Qualitative evaluations from these interventions show positive results. For example, the Girls Power Initiative through its skills training activities economically empowered women to reduce the likelihood of transactional sex. GPI reported that their behaviour change and communication model reduced the likelihood that young women will engage in transactional sex. The Soul City Institute’s interventions were noted to have impacted social norms especially those related to sexual behaviour.

#### Parenting and socialisation

Although socialisation is a key component for the transmission of important social norms[45], there are a few interventions with an explicit focus on improving parent-child relationships (communication and other dimensions of parenting) from sSA. We reviewed two interventions that focused on parenting as a key mechanism of socialization: Families Matter![39] and the Mema Kwa Jamii project (Good Things for Communities project)[41].

Families Matter! was a community-based, group-level intervention that was delivered over five consecutive 3-hour sessions. This intervention was adapted from the USA to a Kenyan context. The goal of the intervention was to reduce sexual risk behaviours among adolescents, including delayed onset of sexual debut, by giving parents the tools they need to deliver primary prevention to their children[38, 39]. Mema Kwa Jamii was a community-based parenting intervention conducted in Tanzania. The intervention aimed to reduce sexual and reproductive health risks among youth by improving parent-child communication including SRH[41, 46].

The pre-post evaluation of Families Matter! showed that the adapted parenting intervention retained its effectiveness, successfully increasing parenting skills in parent-child communication about sexuality and risk reduction. Positive intervention effects at follow-up were found on parenting measures such as: parental attitudes towards sexuality education, and whether children had ever asked their parent about sex issues, parenting practices and parental responsiveness[39]. The intervention also impacted frequency of parent-child sexuality communication from 17% of the children reporting having asked their parent a question about sexuality at baseline to 38% at 12 months follow-up, and 14% of the parents reported being asked a question about sexuality from their child at baseline, to 50% at follow-up. The authors also note that in addition to these positive intervention effects, the programme was also well received by parents who found it beneficial in terms of skills and confidence development. The *Mema kwa jamii* evaluation is ongoing although preliminary results show that parents socialised their children along gender lines.

## Discussion

This review provides a comprehensive narrative summary of fifteen interventions, ten published in peer reviewed publications and five were grey literature. The interventions focused at addressing the economic and social drivers of young people’s risk by exploring pathways of influence. Most of these interventions have been conducted in Southern Africa with limited work in East and West Africa. In this section, we reflect on the interventions by exploring the interventions pathways of influence and highlighting areas for further work.

### A reflection on the interventions and key lessons learnt

Our results indicated that there are numerous interventions targeting adolescent vulnerability in sSA. Most of these interventions target young women in secondary school and those classified as most vulnerable. Many have also concentrated on improving the economic aspect of young people’s life[20, 23, 24, 47] and thus impacting risk in multiple ways. However, although many of the interventions have aimed at the economic pathways to risk and livelihoods opportunities, there has not been much rigorous evaluation of whether the approach works, hence, raising questions as to whether they are actually effective in reducing adolescent sexual risk.

Many interventions may attempt to address multiple structural factors such as social norms, gender, inequality, and poverty and may not necessarily have HIV incidence as the main outcome. Microfinance or microcredit has been used as potential intervention model for HIV/AIDS prevention where rates of infection are high among women and girls who are poor. Such intervention models aim to address gender inequality and empower young women economically thus addressing poverty and gender inequality norms and in the course have a positive effect on outcomes such as condom use, reduction in number of partners, gender violence and transactional sex and ultimately, HIV incidence. Other interventions may focus at addressing social norms that may contribute to risk, while others may focus at addressing poverty/livelihood insecurity with a hope that this would impact sexual risk behaviour. Some interventions have combined an educational component with microcredit and community mobilisation or gender empowerment training combined with life skills and financial literacy. Some address gender inequality and livelihood insecurity through supporting educational attendance for girls.

However, although most interventions aimed at economic empowerment for girls they mention trying to reduce their sexual vulnerability by empowering them economically, the only interventions that had an explicit mention about trying to address risky practices that are partly driven by poverty were: Girls Power Initiative and SHAZ!. Nevertheless, despite the explicit mention of impacting transactional sex behaviour, it is only SHAZ! that was rigorously evaluated for impact. In order to reduce young women’s risk to unintended sexual and reproductive health outcomes, it is important that the drivers of specific risky sexual practices (e.g. transactional sex) are well understood and addressed with appropriate interventions.

There is a speculated link between microfinance and prevention of HIV infection because poverty is a primary structural factor that increases adolescents’ vulnerability to HIV. Most of the interventions that focused on economic empowerment of young women operated with the assumption that increasing young women’s economic circumstances and livelihoods options in the long term would reduce their vulnerability to HIV. Although the goal for providing microfinance[20, 23, 24] and cash transfers is similar[26, 29, 30, 48], the mechanism of action may be different. While microcredit requires financial skills for effective achievement of the goals, cash transfers do not require this skill and thus put less pressure on the young person as there is no repayment of the money. The envisaged mechanisms of action are: meeting basic needs results to less need to engage in transactional sex for survival; and due to reduced economic desperation, young women enhance their ability to negotiate condoms, have fewer partners and avoid much older partners who are typically attractive because of their money. This observation is in line with evidence from the cash transfers intervention[29] and reinforces further the need to tailor specific interventions to specific age-groups.

The interventions on comprehensive sexuality and behaviour change communication have been included because of the possible mechanism of action on social and gender norms which are some of the key structural drivers of young people’s risk. Two of the interventions (Soul Buddyz[35] and Stepping Stones[44]) focused on boys and girls and impacted harmful gender norms such as masculinity and multiple and concurrent partnerships. Transactional sex has been noted to encourage multiple partnerships partly due to the benefits from the exchange that are enhanced when one has many concurrent partners[3, 6, 49]. The outcome from the two interventions point to how interventions that may not have an explicit focus on social structures could still impact them if the right approaches to delivery are employed (e.g. the use of participatory activities) and deserves further exploration.

The role of parents and families in young people’s sexual decision making is gaining prominence. There is considerable evidence showing that family influences, and particularly parenting, have a major influence on young people’s lives and sexual decision making[21, 50]. One of the main pathways in which families and, in particular, parents influence children at a structural level is through the principal mechanism of socialisation[51]. There is a circular relationship between macro-level structural influences and family processes, since parents’ parenting practices are shaped by the wider structural factors and, in turn, reproduce them. As noted by Giddens[52] parenting can therefore be seen as a classic ‘social practice’ that produces, and is produced by, social structures. The main structures reproduced through families that shape vulnerability to HIV include gender, generational hierarchies, economic poverty, systems of cultural beliefs and legal factors[3]. This can be illustrated with respect to gender relationships which are central to sexual health. There are also notable efforts towards theorisation of the role of authoritarian parenting as a structural driver of young people’s risk[53]. Families matter![39] and Mema kwa jamii[40] are examples of interventions that are working at improving parenting and so far although their results are promising, further rigorous work needs to be done to understand how parenting is a structural driver for young people’s risk.

The key lesson learnt from the economic empowerment interventions and as concluded by some of the authors[20, 24] is that microcredit without proper mentorship might not be the best intervention for most adolescents. While girls may grapple with loan repayment, they may also be made vulnerable to HIV infection. However, as noted by the IMAGE study[23], in much older women with already established business skills, microcredit has the potential to reduce poverty while facilitating better health. This implies a need for interventions focused at addressing the structural factors of young people’s SRH risks to have an explicit focus on understanding the pathways of influence.

Another lesson learnt from the interventions is that small loans used for income generation have the potential to reduce poverty directly while also facilitating better health. However, as noted in the TRY intervention[24, 25], and SHAZ![20], economic empowerment interventions with a classic microfinance model may fail with most vulnerable girls because their main needs may not initially be entrepreneurship but for physical safety, social support, friendship, and mentorship. Lessons from the TRY intervention pointed to the need to first give girls these important skills, and then would they be able to think constructively about making and saving money. Given these important lessons from both TRY and SHAZ! interventions, a staged approach to livelihood interventions is more promising as it takes into account the evolving capabilities and vulnerabilities of girls and young women. SHAZ! further pointed to the additional benefit of enhancing social support from families and friends as necessary inputs to strengthen adolescent girls’ individual sense of economic empowerment.

Achievements from interventions that focus on improving school attendance point to the benefits of reducing economic barriers to school attendance for girls. The combination of safe spaces, financial education, and savings accounts addresses a range of vulnerabilities that girls face including social isolation, financial responsibilities, and relationships with men that involve high levels of economic dependence. These interventions point to the need to focus on sensitive and deep rooted social norms determining relationships if these have to be modified and giving attention to the wider community and not just young women. This is crucial for obtaining community buy-in. Many parents need support to effectively define and convey their values and expectations about sexual behaviour and communicate important messages about HIV, STIs and pregnancy prevention with their children.

### Intervention design and evaluation

This review points to interesting methodological lessons that scholars interested in improving young people’s SRH could learn from. One important lesson is the requirement for flexibility in design and the use of a phased approach in interventions and programmes for vulnerable young people. This approach was utilised and found to be useful in two of the programmes (SHAZ![20] and TRY[24]) and could offer useful insights for structural interventions with young people. We however acknowledge that although such an approach may be interesting, it may be challenging to get funding and ethical approval for it due to a lack of clarity on design from the outset.

Evaluation is an important component of any intervention activity in order to gauge whether it was able to achieve what it set out to achieve. Evaluation approaches for the interventions reviewed varied from one intervention to another with others not specifying the methodology used to evaluate their intervention but reporting on results. The lack of a rigorous evaluation would be attributable to factors such as the funding agency requirements, the capacity of the intervention implementation team and available funds for such an evaluation. This is an area that needs lots of strengthening if researchers and those in programme work have to effectively engage with each other, avoid unnecessary duplication of effort and achieve the ultimate goal of improved sexual and reproductive health among young people through structural interventions.

Although there is lots of interesting interventions targeted at young people, it is evident from the review that there is substantial variation in design and methods of evaluation, effectively limiting the conclusions that can be drawn. Due to the heterogeneity of the interventions included in this review, we are not in a position to comment on intervention effectiveness. Eight of the interventions utilised a randomised control design. Others do not explicitly mention the design they employed. This review has the following limitations: the inclusion of both published and unpublished literature although advantageous in many ways, it also created challenges as most of the unpublished work had not been rigorously evaluated. In addition, we did not include interventions that did not have written documentation (i.e. reports, briefs, publications) on their work. This was mainly because of the difficulty referencing such interventions but also due to potential for bias in reporting such work. We acknowledge that this exclusion might have left out interesting work on young people’s vulnerability from the region. It is also crucial to consider the methodological limitation of the interventions themselves. The review has included interventions that employed different designs and some that did not clearly define their design. In as much as we may acknowledge this as a potential limitation, we would like to point out that our objective was not to compare the effectiveness or impact but to describe the interventions and provide a starting point at which policy makers and researchers would use for prioritization.

## Conclusion

Whilst the number of interventions aimed at addressing the structural drivers of risk among young people are on the rise, there is a need for interventions to have a clear focus, indicate the pathways of risk they are trying to address and have a rigorous evaluation strategy to assess whether and how they work to reduce young people’s vulnerability to HIV.

## Authors’ original submitted files for images

Below are the links to the authors’ original submitted files for images. Authors’ original file for figure 1
