# Supplementary material for: A review of interventions addressing structural drivers of adolescents’ sexual and reproductive health vulnerability in sub-Saharan Africa: implications for sexual health programming
Source: Reprod Health. 2014 Dec 13;11:88. doi: 10.1186/1742-4755-11-88 (PMC4290135; doi:10.1186/1742-4755-11-88)
Supplement: Supplementary file 1 — Authors’ original file for figure 1 [file 12978_2013_340_MOESM1_ESM.doc]

**Table 1:** DESCRIPTIONS of **interventions for young people**

| **Intervention/country** | **Aim** | **Intervention method/design & Duration** | **Target group &Sample size** | **Outcomes/results** |
| --- | --- | --- | --- | --- |
| **Economic empowerment of women** | | | | |
| 1) Shaping the Health of Adolescents in Zimbabwe (SHAZ! )Program [1] | Increased knowledge  Increased economic empowerment  Reduced inter-generational TS | **Pilot study**  -Uncontrolled study for 6 months  - Microcredit loans  - Business skills training  -Mentorship  **Phase II Study:**  Randomized clinical trial (RCT)  Study  Duration 24 months,  Adaptation of Stepping  Stones, including  expanded training  including negotiation  skills, Integrated social support  Access to HIV and  reproductive health  services | - 50 poor orphaned, out-of-school,   girls aged 16-19 years   - Living on the outskirts of Harare, Zimbabwe   - 315 aadolescent girls, orphans,  average age 18 | - Increase in HIV-related knowledge and relationship  - power, no significant change in current sexual activity  or condom use at last sex  -Increased relationship power [49]  - Increased HIV risk through new mobility and economic  strategies  -Increase in HIV-related knowledge and relationship power, no significant change in current sexual activity or condom use at last sex  - Decrease in food insecurity  - Increase in equitable gender norms  - Physical and sexual violence reduce by 58% over a 2-year period |
| 2) Intervention with Microfinance for AIDS & Gender Equity ( IMAGE) [2, 3], South Africa | Reduced HIV risk behaviour | Cluster randomised trial, duration of 3 year   - Microfinance (individual borrowing and repayment of loans over 10 or 20 week cycles) - Participatory learning and action curriculum integrated into loan meetings (10 training sessions done within centre meetings every 2 weeks (approx. 6 months)) Community mobilization for 6 to 9 months following initial training - HIV prevention education | - A sample of 430 poor women aged 14-35 years identified through participatory wealth ranking | - 55% increase in experience of IPV after 1 year  - Increase in HIV knowledge, communication, testing & risk reduction  - 32% reduction in communication with household members to young people in households  - Greater involvement in collective action and social groups  -No impact on HIV incidence in wider community  - No difference in unprotected sex at last  occurrence with non-spousal partner in past 12 months  - 11% increase in condom use at last sex |
| 3) The Tap & Reposition Youth (TRY) [4, 5],  Kenya | Increased reproductive health & HIV knowledge  Increased sexual negotiation skills  Increased Income & savings | Pre-test, post-test design, with matched comparison (222 pairs), length of participation ranged from <1 year (n=71), 1 to 2 years (n=81) and 2 to 3 years (n=70)   - Group-based microfinance loans, Livelihoods skills training - RH & HIV prevention training | - A total of Out-of-school   females aged 16-22 years   - Living in low income & slum areas of Nairobi | - Increase Savings  - increase in liberal attitudes towards gender roles  - 1.7 times more likely to refuse sex than girls in control group  - 3 times more likely to insist on condom use than girls in control group |
| 4) Incentivising safe sex: a randomised trial of conditional cash transfers for HIV and sexually transmitted infection prevention in rural Tanzania [6] | To evaluate the use of conditional cash transfers as a HIV and sexually transmitted infection prevention strategy to incentivise safe sex | An unblended, individually randomised controlled trial   - Intervention arms: low value conditional cash transfer v.s., high value conditional cash transfer - Tested participants every 4 months over a 12 months period for the presence of common STIs | - A sample of 2399 persons aged 18-30 years | - High value CCT arm v.s. controls: adjusted RR = 0.073 (95% CI 0.47-0.99) - High value CCT arm v.s., low value CCT arm: RR = 0.76 (95% CI0.49 -0.92) - Significant reduction in the combined point prevalence of four curable STIs among high value CCT arm |
| 5) Survival skills training for orphans (SSTOP)  [7], Mozambique | To reduce transactional sex | Intervention:  Income generating skills   - Girls aged 9-13 learned to make soap, candles, sewing, or knitting - Girls aged 14-19 attended sewing classes; HIV prevention education; & gender training including legal protection for women | - Females aged 14-19 years - Responsible for caring for younger siblings, & other disadvantaged girls | Qualitative & anecdotal evidence found:   - Increased financial organization - Increase vocational skills - Reduction in early marriage - Increased economic empowerment - Reduction in early sexual activity without protection |
| Creating futures[8] (Durban, South Africa) | Objective 1:  To strengthen young people’s livelihoods and economic power through reflection and action  Objective 2: Aimed to reduce women’s experience and men’s perpetration of physical or sexual IPV | Pilot intervention combining Stepping Stones and Creating Futures  -The study design was an interrupted time-series design, with baseline measures at zero and two weeks and follow-ups at six and 12 months post-baseline.  -Consisted of livelihoods and economic power intervention involving 21 sessions of three hours, delivered by trained peer facilitators | - Piloted in urban informal settlements in with 232 young people (110 men, 122 women) - Average age of 21.7 years | **Objective 1:**  - Livelihoods improved for women and men after the intervention  -Mean earnings in the past month increased over the 12 months. For women this increased from US$14 at baseline to US$49 (a 345% increase (p<0.0001)) at 12 months and for men from US$36 at baseline to US$104 (a 283% increase (p<0.0001)) at 12 months  **For objective 2:**  Women reported a statistically significant reduction in their experience of sexual or physical IPV in the past three months from 29.9% at baseline to 18.9% at 12 months (a 37% reduction (p<0.046)  -women’s experience of sexual IPV also declined significantly from 11.1% at baseline to 3.6% at 12 months (p<0.018)  -Men’s perpetration of physical or sexual IPV in the past 3 months, while declining from 25% to 21.9% (a 23% reduction) was not statistically significant |
| **Economic empowerment plus school Attendance** | | | | |
| 6) Zomba cash transfer [9], Malawi | Increased income  Increased Education  Reduce HIV risk | Randomised control trial, 2 years  Cash transfers (CTs) conditional and on regular school attendance v.s. unconditional CTs (average amount US $10) | A sample of 1289 never married girls aged 13-22 years in 176 enumeration areas in Zomba | **One-year follow-up:**  - Reduced onset of sexual activity by 31.1%  **At 18 months follow-up:**  -Intervention group had 64% reduction in HIV  prevalence and 76% reduction in HSV-2  prevalence  - Reduced age of partners in those in  intervention  - No significant differences between conditional and unconditional intervention group, although the study was not powered to show this |
| 7) Western Kenya schooling intervention [10] | To reduce HIV incidence in schools | Randomised control trial, 4 years,  Comparing 4 school-based HIV/AIDS interventions:   - Training teachers in HIV/AIDS curriculum - Critical thinking on role of condoms - Reducing the cost of education by providing school uniforms - Relative risk campaign | A sample of 70,000 school boys and girls in school | Teacher training:  - no impact childbearing  - Increase in HIV knowledge  If pregnant, more likely to be married  Critical thinking:  - Increase knowledge & condom use  -No impact on sexual activity  School uniforms:  - Reduction in dropout rates 17% in boys, 14% in girls  - Reduction in teen marriage 9% in girls  - Reduced childbearing 12%  Relative risks:  - Reduction in childbearing 28%  - Increased sexual activity in boys  - No impact on pregnant teen couples  - Reduction in cross-generational pregnancies 61% |
| **Gender empowerment and safe spaces for young people** | | | | |
| 8) Binti Pamoja Centre (Daughters United centre) [11, 12], Kenya | Create safe spaces for girls to reduce: violence, Female genital mutilation, Sexual abuse, Rape, Prostitution Poverty and Increase: Reproductive health knowledge, Financial education, Leadership & personal skills | Community intervention:   - Sampled adolescents from 4 ethically distinct villages in Kibera - Mapped all safe spaces in the community - Used photography, drama, writing & group discussion - Peer education & empowerment workshops - Developing skills in budgeting, savings, setting financial goals - Provided educational scholarships | Girls aged 11-18 living in the Kibera slum | **2002 to present**   - Baseline data highlights social isolation for many girls & 55% of girls live with neither or only one parent - >30 safe spaces established reaching >1000 girls - Positive changes in social networks, mobility & gender norms - Increased financial literacy, banking services usage, savings, & communication with parents/guardian on financial issues - Increased confidence & positive self-esteem |
| 9) Siyakha Nentsha [13], South Africa | A life-orientation program to improve lifelong skills & well being of young people | Quasi-experimental, control  arm, 18 month  follow-up, 4 years  Three study arms:   - SRH/HIV, social support, financial education - SRH/HIV & social support - Delayed Intervention (i.e. control group) | Boys & girls aged 14-16 in schools | - Increased autonomy for girls in how they spend their money & control their lives - Increased HIV related knowledge - Young men had reduced onset of sexual activity and fewer partners |
| 10) ICRW Vitu Newala [14], Tanzania | Understand specific vulnerabilities of adolescent girls and empower them, increase girls positive attitudes and beliefs on girls’ social protection | Pilot project  Qualitative assessments throughout:   - Repeating the same participatory learning activities, - Series of IDIs with young people, - An evaluation workshop | Adolescent girls | - Video parlours, discos & traditional initiation ceremonies identified as places where girls felt unsafe - Community put in place laws & changed practices to provide social protection |
| **Comprehensive sexuality and Behaviour change communication** | | | | |
| 11) Soul City Institute for health & development [15],  South Africa | Increase: social change, Social mobilization, Advocacy and reduce HIV incidence | Promoting health & social change via TV, radio, & print  Soul Buddyz:   - Spin off of Soul City TV series using edutainment   One love:   - Challenged social norms on multiple & concurrent partnership | Soul buddyz:   - Children aged 8-14 years, their teachers & their caregivers - One love: Adults | Soul City & Soul Buddys exposure   - Increased: Self-perceptions on risk, Resistance to peer pressure - Statically significant shifts in social norms, especially sexual norms - Reduced Perception of women’s dependence on men (68% vs. 61%, p<0.05) |
| 12) Stepping Stones [16, 17], South Africa | Increase: Sexual health knowledge, Communication skills, Ccritical reflection and reduce Sexual health risk | Cluster randomised controlled trial, 2 years   - 70 villages randomized to either 13 3-hour sessions and 3 peer group meetings, or a 3-hour session on safer sex and HIV. | A sample of 1077 HIV negative Persons aged 15-26 years, mostly attending school | - HIV IRR = 0.85 (95%CI: 0.60, 1.20; p=0.35) - HSV2 IRR = 0.69 (95%CI: 0.47, 1.03; p=0.07) - Men’s disclosure of perpetrating severe Intimate partner violence reduced at 12 & 24 months (p=0.11 & p=0.05) - Reduced Problem drinking among men |
| **Parenting and Socialisation** | | | | |
| 13) Families Matter! (FMP), [18, 19], Kenya | Reduce age at first sex and increase ppositive parenting practices | Community-based intervention using parent-child dyads, 2 years (2004-2006)  Five consecutive 3-hour sessions on sexual risks and effective parent-child communication | 375 Parents/carers of 10-12 year-olds | - Increased Parenting skills & communication about sexuality & risk reduction  -Parents’ attitudes regarding sexuality education changed positively.  Five of the six composite parenting scores reported by parents, and six of six reported by children, increased significantly at 1 year post-intervention. |
| 14) Mema kwa Jamii (Good Things for Communities, *MkJ)*, [20, 21], Tanzania | Reduce SRH risks in youth through improved parenting | Community-based pilot parenting intervention, 2007-2010  Opinion leaders in four communities trained to training peer parents on parenting following diffusion of innovation theory over a period of 1 year | Approximately 1355 parents of young people aged 10-18 years | Qualitative indications of impact on:  -Parents socialised their male children differently from female  -Improved parent-child relationships and collective efficacy |

1. Dunbar MS, Maternowska MC, Kang MS, Laver SM, Mudekunye-Mahaka I, Padian NS: **Findings from SHAZ!: a feasibility study of a microcredit and life-skills HIV prevention intervention to reduce risk among adolescent female orphans in Zimbabwe**. *J Prev Interv Community* 2010, **38**(2):147-161.

2. Pronyk PM, Hargreaves JR, Kim JC, Morison LA, Phetla G, Watts C, Busza J, Porter JD: **Effect of a structural intervention for the prevention of intimate-partner violence and HIV in rural South Africa: a cluster randomised trial**. *Lancet* 2006, **368**(9551):1973-1983.

3. Pronyk PM, Kim JC, Abramsky T, Phetla G, Hargreaves JR, Morison LA, Watts C, Busza J, Porter JD: **A combined microfinance and training intervention can reduce HIV risk behaviour in young female participants**. *AIDS* 2008, **22**(13):1659-1665.

4. Erulkar A, Bruce J, Chong E, Dondo A, Sebstad J, Matheka J, Banu KA, Gathuku A: **Tap and Reposition Youth (TYR): providing social support, savings and microcredit opportunitites for yougn women in areas with high HIV prevalence.** In: *Promoting Healthy, Safe and Productive Transitions to Adulthood Brief no15.* New York: Population Council; 2006.

5. Hall J, Dondo A, Sebstad J: **Tap and Reposition Youth (TRY) Program: Providing Social support, savings and microcredit opportunities to adolescent girls at risk for HIV/AIDS in Kenya**. In: *Microfinance Brief.* vol. http://www.popcouncil.org/pdfs/MicrofinanceBrief_TRY.pdf. New York: The Population Council Inc.; 2006.

6. De Walque D, Dow WH, Nathan R, Abdul R, Abilahi F, Gong E, Isdahl Z, Jamison J, Jullu B, Krishnan S *et al*: **Incentivising safe sex: a randomised trial of conditional cash transfers for HIV and sexually transmitted infection prevention in rural Tanzania**. *BMJ Open* 2012, **2**:e000747.

7. TIOS: **Training Internationally for the orphans and their survival**. In*.* Avenida Samora Machel, Chioio: TIOS; 2008: http://aosci.tios@gmail.com.

8. Gibbs A, Jewkes R: **Stepping Stones and Creating Futures Intervention: Outcomes of a behavioural and structural pilot intervention to build gender equality and economic power among young people in urban informal settlements in South Africa. Project Brief**. In*.*, vol. http://www.heard.org.za/gender/creating-futures-stepping-stones: HEARD, MRC South Africa, Project Empower, University of KZN; 2013.

9. Baird SJ, Garfein RS, McIntosh CT, Ozler B: **Effect of a cash transfer programme for schooling on prevalence of HIV and herpes simplex type 2 in Malawi: a cluster randomised trial**. *Lancet* 2012, **379**(9823):1320-1329.

10. Duflo E, Dupas P, Kremer M, Sinei S: **Education and HIV/AIDS prevention: Evidence from a randomized evaluation in Western Kenya**. In*.* Edited by paper WBPw: World Bank; 2006.

11. Global giving: **"Empowering girls in Kibera"**. In*.*, vol. https://www.globalgiving.com/pr/1800/proj1771a.html; 2008.

12. Carolina for Kibera: **Tuuangane Tuangaze (Let's unite and shed light). 2006 annual report**. In*.* Chapel Hill, NC; 2007.

13. Hallman K, Roca E: **Siyakha Nentsha: Building economic, health, and social capacities among higly vulnerable adolescents in KwaZulu-Natal, South Africa**. In*.*, vol. Brief no. 4: Population Council; 2011.

14. McCleary-Sills J, Douglas Z, Rwehumbiza A, Hamisi Z, Mabala R: **Vijana Twaweza Newala:Findings from a participatory Research and Action Project in Tanzania**. In*.* Washington DC; 2011.

15. Goldstein S, Usdin S, Scheepers E, Japhet G: **Communicating HIV and AIDS, what works? A report on the impact evaluation of Soul City's fourth series**. *J Health Commun* 2005, **10**(5):465-483.

16. Jewkes R, Wood K, Duvvury N: **'I woke up after I joined stepping stones': meanings of an HIV behavioural intervention in rural South African young people's lives**. *Health Education Research* 2010, **doi:10.1093/her/cyq062**.

17. Jewkes R, Nduna M, Levin J, Jama N, Dunkle K, Wood K, Koss M, Puren A, Duvvury N: **Evaluation of stepping stones: a gender transformative HIV prevention intervention** In*.*, vol. htt://www.mrc.ac.za/policybriefs/stepping stones.pdf: Medical Research Council, South Africa; 2007.

18. Poulsen MN, Vandenhoudt H, Wyckoff SC, Obong'o CO, Ochura J, Njika G, Otwoma NJ, Miller KS: **Cultural adaptation of a U.S. evidence-based parenting intervention for rural Western Kenya: from parents matter! To families matter!** *AIDS Educ Prev* 2010, **22**(4):273-285.

19. Vandenhoudt H, Miller KS, Ochura J, Wyckoff SC, Obong'o CO, Otwoma NJ, Poulsen MN, Menten J, Marum E, Buve A: **Evaluation of a U.S. evidence-based parenting intervention in rural Western Kenya: from parents matter! To families matter!** *AIDS Education Prevention* 2010, **22**(4):328-343.

20. Remes P, Bazil V, Komrower J, Nyalali K, Charles J, Wight D, Changalucha J, Obasi A: **"We're surpassed by our children!" Parents and caregivers demand parenting interventions to support HIV/STI in rural Mwanza, Tanzania**. In: *International AIDS conference* Edited by IAS. Mexico city; 2008.

21. Remes P, Wight D: **"Nowadays People Use Psychology, We didn't use that we just grew up": Exploring Contemporary Parenting in Rural Mwanza, Tanzania**. *Journal of Adolescence Research* 2011, **Under Review**.
